# One-Step Release Technique for Tendon Extraction During Biceps Tenodesis

**DOI:** 10.1016/j.eats.2023.09.020

**Published:** 2024-01-01

**Authors:** Emanuele Maggini, Markus Scheibel

**Affiliations:** a Department of Medical and Surgical Specialties, Radiological Sciences, and Public Health, University of Brescia, Piazza del Mercato, Brescia, Italy; b Department of Shoulder and Elbow Surgery, Schulthess Clinic, Zürich, Switzerland; c Center for Musculoskeletal Surgery, Campus Virchow, Charité-Universitaetsmedizin Berlin, Berlin, Germany

## Abstract

All biceps tenodesis techniques involving extra-articular tendon preparation consist of 2 distinct steps for tenotomy and tendon grasping. These 2 maneuvers are performed by different instruments. A single-step tendon release technique is described for both arthroscopic and open shoulder surgery. This technique finds application in arthroscopic proximal suprapectoral tenodesis, open treatment of proximal humerus fractures, and suprapectoral mini-open tenodesis. Herein, we describe an arthroscopic biceps tenodesis technique that combines the actions of cutting and gripping the tendon in a single step.

Pathology of the long head of the biceps tendon (LHBT) can be a pain generator.^1^, ^2^, ^3^ There are then 2 possibilities for LHBT tendinopathy: a simple tenotomy or a tenodesis. The choice among the 2 techniques is based on several factors, including the patient’s age and functional demands, the condition of the biceps, and concerns regarding residual cosmetic deformity after biceps tenotomy.^2^^,^^4^^,^^5^

Tenodesis of the LHBT is technically more challenging than a simple tenotomy. There are several popular procedures for performing biceps tenodesis by arthroscopic, mini-open, and open techniques.^1^^,^^2^ So far, in all the techniques in which the tendon is prepared extra-articularly, there are 2 separate steps to perform LHBT tenotomy and to exteriorize the tendon. In general, there is not a singular instrument that completes 1 task. This reduces clinical efficiency and increases procedural time. The BITER (BIceps TEndon Releaser; KARL STORZ SE & Co. KG) is an instrument specifically designed for cutting and grasping, which allows in 1 step to tenotomize and pull extra-articularly the LHBT.

Herein, we report the technique as previously published,^6^ describing simultaneous resection and grip of the biceps (Video 1). The intent is to describe a technique that simplifies a central step in LHBT tenodesis, providing a more effective and rapid procedure.

## Surgical Technique

### Indications

• Arthroscopic proximal suprapectoral tenodesis
• Suprapectoral mini-open tenodesis
• Open treatment of proximal humerus fractures

### Contraindications

• Subtotal tears or tears with loss of substance
• Subpectoral tenodesis

The patient is placed in the beach-chair position and the surgical arm is secured in an arm-holding device. The entire scapula and arm are prepared and draped, and bony landmarks are drawn on the shoulder. This surgical technique for arthroscopic proximal suprapectoral tenodesis with knotless anchor fixation requires 4 steps.

### Step 1: Glenohumeral Exploration

The 4-mm 30° scope is introduced in the standard posterior viewing portal, and the glenohumeral joint is explored. The LHBT is evaluated from its glenoid origin medially and followed into the bicipital groove laterally. Pathology of the biceps tendon is confirmed: instability, inflammation, degeneration, and tears are noted.

### Step 2: Tenotomy and Exteriorization of the LHBT

**Fig 1:**
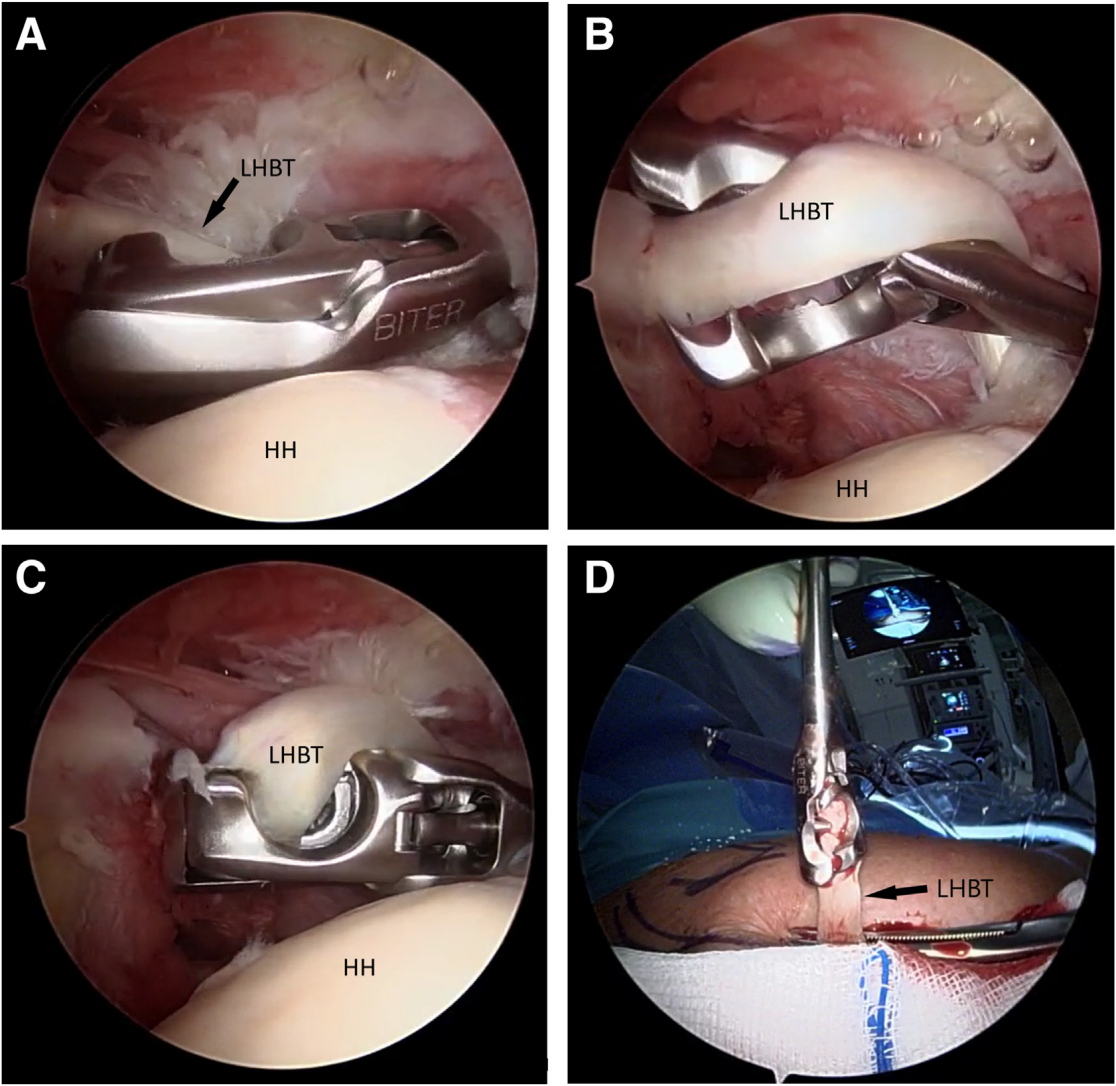
Right shoulder. Beach-chair position. Posterior view. (A) Introduction of an instrument designed for cutting and grasping (BITER) into the glenohumeral joint by the anterolateral portal. (B) Twisting of the LHBT around the instrument and engagement. (C) Tenotomy: the BITER’s blade cuts the tendon and the tip pierces it, keeping its grip. (D) The BITER extracted from the joint still attached to the biceps. (HH, humeral head; LHBT, long head of the biceps tendon.)

If the LHBT is determined to require tenodesis, an anterolateral portal is established with an outside-in technique using a spinal needle. The BITER is introduced by the anterolateral portal (Fig 1A). By turning the instrument, the tendon engages in the housing (Fig 1B). By tightening it, a spike transfixes the tendon holding it in place while a blade precisely cuts the end (Fig 1C). The biceps tendon is tenotomized close to its origin, pulled extra-articularly, and exteriorized (Fig 1D). The penetration of the LHBT by the spike of the BITER allows the tendon to be pulled out directly without any additional steps.

### Step 3: Tendon Preparation

**Fig 2:**
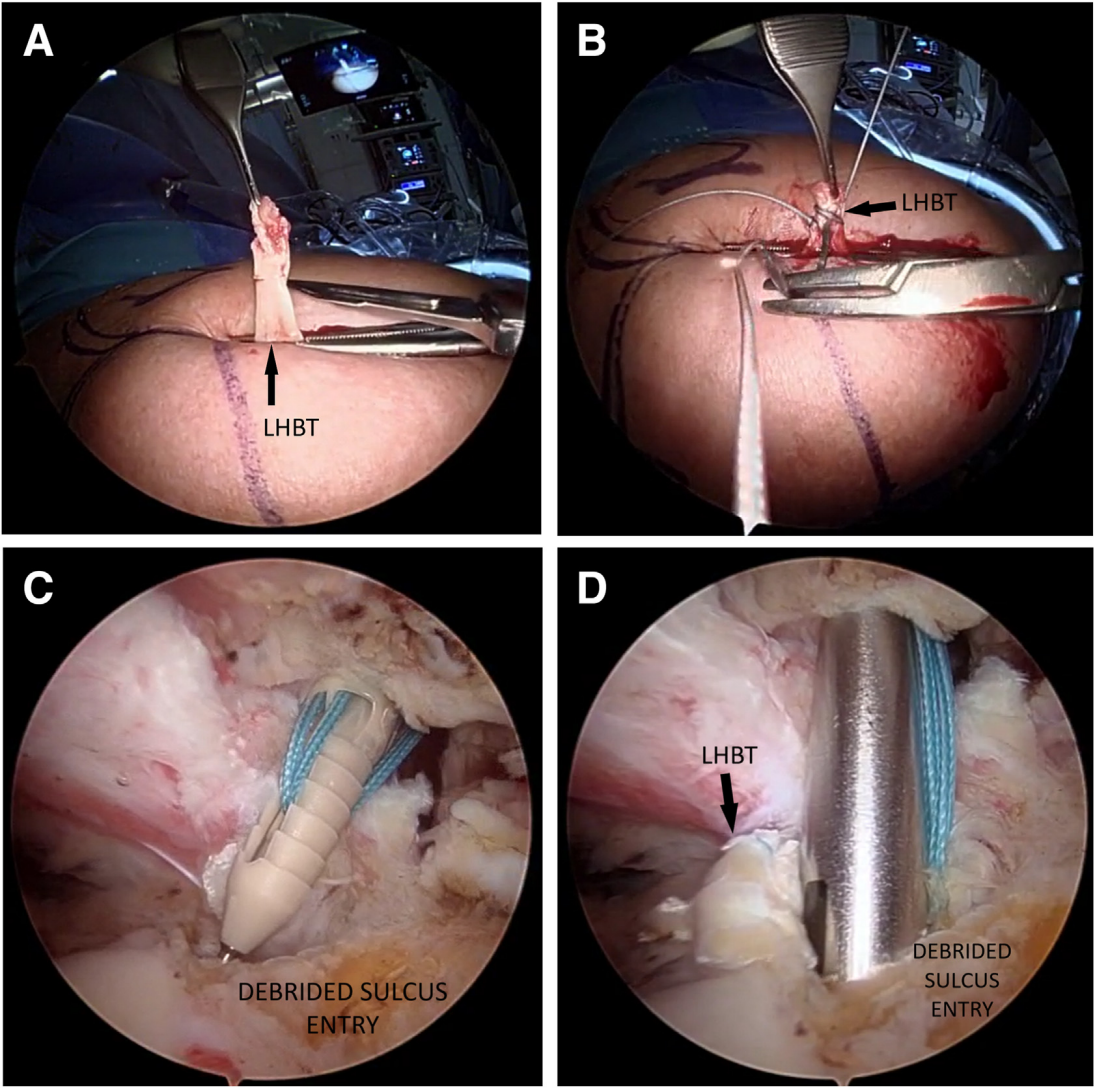
Right shoulder. Beach-chair position. (A) Exteriorization and resection of the proximal part of the tendon. The LHBT is secured to a clamp so not to lose it. (B) Tendon stump suture with the baseball suture technique using a No. 2 FiberWire suture. (C) The knotless PEEK anchor loaded with the 2 tails of No. 2 FiberWire before being pushed into the bone at the sulcus entry. (D) Fixation of the LHBT at the insertion site with the knotless anchor. (LHBT, long head of the biceps tendon.)

The biceps is secured to a Kocher clamp so not to lose it. The proximal part of the tendon is resected and evened to approximately 1.5 cm (Fig 2A). The stump is reinforced with a No. 2 FiberWire suture (Arthrex) using the baseball stitch technique (Fig 2B).

### Step 4: Knotless Anchor Fixation

A debridement of the reinsertion zone at the sulcus entry is made. The area is prepared with a motorized shaver.

An onlay anchor tenodesis using a self-punching knotless anchor (ReelX STT; Stryker Corp) is performed. The PEEK anchor is loaded with the 2 tails of No. 2 FiberWire sutures. While maintaining slight tension on the suture limbs, the anchor is slid down to the bone at the insertion site (Fig 2C). The anchor is hammered until it is flush with the cortical surface of the bone (Fig 2D). The suture threads are pulled until the desired suture tension is obtained, adding a tensile load to the compression of the tendon on the footprint. During this maneuver, how the tendon rises on the anchor can be seen. The tether suture is released from the inserter handle and the suture threads are cut.

## Discussion

Surgical treatment for pathologies of the LHBT is limited to removal of the intra-articular portion of the tendon, with either tenotomy or tenodesis. Both procedures have advantages and disadvantages. LHBT tenotomy is simple but, in some cases, can lead to a cosmetic Popeye sign deformity and subjective loss of strength for elbow flexion and supination.^2^, ^3^, ^4^^,^^7^^,^^8^

Tenodesis of the LHBT is technically more challenging than simple tenotomy. In LHBT tenodesis, there is a differentiation between an open, a mini-open, and an arthroscopic procedure in the type of fixation and in the choice of fixation implants. Whatever technique is adopted, so far there are 2 distinct steps to tenotomize and exteriorize the LHBT.

This article describes an arthroscopic tenodesis in the proximal-suprapectoral position that combines the actions of cutting and grasping the tendon in a single step. Typically, there are 2 distinct steps for tenotomy and tendon gripping. In fact, these 2 maneuvers are performed by different instruments. Usually, a preliminary first step is intra-articular transfission of the LHBT at its entrance into the groove using a spinal needle. This will prevent the tendon from retracting into the groove in the transition between tenotomy and tendon grasping. Tenotomy can be performed with a knife, an arthroscopic scissor, a punch, or electrocautery. After being cut, the tendon is grasped firmly at the proximal part while the spinal needle is removed. The task can be accomplished with a clamp or an arthroscopic grasper. At this point, the LHBT can be exteriorized to continue with the next steps. The key goal achieved using this technique is a simplification of this stage of the surgery. The BITER was developed as an alternative to conventional instrumentation. Combining the action of cutting and grasping, this device provides the function of several other standard instruments. In 1 step, the LHBT can be resected, gripped, and pulled out, without the need for temporary fixation with a spinal needle and without the need to change instruments. There is a model of this instrument designed for the right shoulder and one for the left shoulder, so that the tendon is engaged by making a rotation. Listed in Table 1 are some of the pearls and pitfalls.

**TABLE I:** Pearls and Pitfalls for the 1-Step Release Technique for Tendon Extraction During Biceps Tenodesis

Pearls	Pitfalls
Always debride the sulcus entry before inserting the LHBT.	Grasping a LHBT that shows loss of substance may result in tearing and necessitate a subpectoral technique.
Use of a self-punching expandable anchor maximizes compression of the biceps against the prepared bony surface of the footprint.	The top of the tenodesis anchor should be flush with the cortical bone. Insertion too shallow could result in pullout, impingement, or irritation; insertion too deep could weaken fixation strength.

LHBT, long head of the biceps tendon.

In addition to this simplification of the procedure, the proposed technique has other advantages (Table 2). This technique is suitable for both arthroscopic and open shoulder surgery: in particular, it can be applied to the osteosynthesis of the proximal humerus fracture. In this situation, the LHBT tendon is at risk of injury because of its proximity to fracture fragments and to the osteosynthesis plate. In case of doubt, a tenotomy of the intra-articular portion of the LHBT and, if necessary, a soft tissue tenodesis distal to the intertubercular sulcus should be performed.^2^ It may happen that the rotator interval is intact, and access to the supraglenoid tubercle may not be easy. The BITER makes it easier to reach the LHBT in such cases.

**TABLE II:** Advantages, Disadvantages, and Limitations of the 1-Step Release Technique for Tendon Extraction During Biceps Tenodesis

Advantages	Disadvantages and Limitations
Eliminates additional steps and tools, speeding and simplifying the procedure	Not indicated in the case of subtotal LHBT tears or tears with loss of substance
Suitable for both arthroscopic and open shoulder surgery	Not indicated in the case of subpectoral tenodesis
Minor soft tissue damage	The intratubercular LHBT segment is not resected

LHBT, long head of the biceps tendon.

Finally, an additional advantage of this arthroscopic technique, in comparison with mini-open techniques, is less soft tissue damage.

However, there are situations in which this technique cannot be applied. First, it is not indicated in the case of subtotal tears or tears with loss of substance. Under such conditions, the BITER in grasping the tendon could tear it; moreover, given the poor quality of the tendon, proximal fixation does not provide an adequate strength. In addition, the technique is not indicated in the case of subpectoral tenodesis, where the LHBT stump is exteriorized more distally.

The disadvantage of proximal suprapectoral tenodesis is that the intratubercular part of the LHBT, which often has synovitic changes, is not resected. To date, however, it has not been possible to prove or definitively rule out that this portion continues to cause symptoms after static fixation by tenodesis.

For the procedures in which this technique finds application, results have already been published. Scheibel et al.^6^ compared the clinical and structural outcome after arthroscopic soft tissue tenodesis vs an arthroscopic bony fixation anchor tenodesis. The bony fixation tenodesis was performed with a suture anchor or a knotless anchor; in the latter case, the technique used is similar to that described in this article. The results showed that anchor fixation provides significant advantages concerning the clinical and structural outcome. Kerschbaum et al.^9^ evaluated the clinical results of a simultaneous biceps tendon treatment (tenotomy or tenodesis) during plate osteosynthesis of proximal humerus fractures. They found that the simultaneous surgical treatment of the LHBT during plate osteosynthesis of proximal humeral fractures brings good clinical and cosmetic results.

In conclusion, the BITER can be a useful device for both arthroscopic and open shoulder surgery. The technique described, by performing a single-step LHBT release, results in a faster and more efficient procedure.

## Supplementary Data

ICMJE author disclosure forms

Video 1 Video of an arthroscopic 1-step release technique for tendon extraction during biceps tenodesis. Right shoulder. Beach-chair position. Diagnostic arthroscopy and creation of an anterolateral portal with an outside-in technique. Introduction of the BITER by the anterolateral portal. Tenotomy using the BITER and exteriorization of the tendon stump. Resection of the proximal part of the tendon and reinforcement of the stump with a No. 2 FiberWire suture using the baseball stitch technique. Debridement of the reinsertion zone at the sulcus entry with a motorized shaver. Long head of the biceps tenodesis using a self-punching knotless anchor loaded with the 2 tails of No. 2 FiberWire sutures. Tensioning of the suture threads, release of the tether suture from the inserter handle, and cutting of the suture threads. A highly stable long head of the biceps tenodesis is achieved.
